# *Murdannia saddlepeakensis* (Commelinaceae) – a new species from Andaman and Nicobar Islands, India

**DOI:** 10.3897/phytokeys.20.3611

**Published:** 2013-02-08

**Authors:** M. Venkat Ramana, Mayur Nandikar, R. V. Gurav, Johny Kumar Tagore, M. Sanjappa

**Affiliations:** 1 Botanical Survey of India, Andaman and Nicobar Regional Centre, Haddo, Port Blair – 744 102, Andaman and Nicobar Islands, India; 2 Department of Botany, Shivaji University, Kolhapur; 3 Former Director, Botanical Survey of India, Kolkata, India

**Keywords:** Commelinaceae, *Murdannia saddlepeakensis*, new species, Andaman and Nicobar Islands, India

## Abstract

*Murdannia saddlepeakensis* (Commelinaceae), a new species from the Andaman and Nicobar Islands, India, is described and illustrated. The new species is remarkable for its narrowly linear leaves, two fertile stamens, single seeded locule and scorbiculate seeds.

## Introduction

The genus *Murdannia* is represented by 54 species (Govaerts and Faden 2004), of which 27 taxa are reported from India (modified after Karthikeyan et al. 1989). Three new taxa viz., *Murdannia fadeniana* Nampy & Joby (Nampy and Joby 2003), *Murdannia satheeshiana* Joby et al. (Joby et al. 2011) and *Murdannia brownii* Nandikar & Gurav (Nandikar and Gurav 2011) have been described during the last decade. In addition, *Murdannia striatipetala* Faden has been rediscovered from India (Nandikar et al. 2011). The Western Ghats of India alone comprises twenty-two species and serve as a major centre of diversification for *Murdannia*.

The Andaman and Nicobar Islands harbour luxuriant lowland rainforests besides wetlands, mangroves and coral reefs. The floral elements of these Islands often show close affinity with that of Indonesia, Malaysia, Myanmar, Thailand and Sri Lanka. Saddle Peak National Park which is located in the North Andaman Islands harbours unique stunted evergreen vegetation that is found only in restricted localities of the Andaman Islands (Rao 1986).

During a recent botanical excursion, we came across an interesting specimen of *Murdannia* in an open scrub forest of Saddle Peak National Park. The specimens were collected and critically studied. It was found that the specimens did not match any of the known species of the genus and hence have been described and illustrated here as a novelty. In addition, a key for *Murdannia* species of Andaman and Nicobar Islands and some other species similar to *Murdannia saddlepeakensis* has been provided to facilitate identification.

## Taxonomic treatment

### Murdannia saddlepeakensis

M.V.Ramana & Nandikar sp. nov.

urn:lsid:ipni.org:names:77124862-1

http://species-id.net/wiki/Murdannia_saddlepeakensis

Figs 1 , 2

#### Type.

**Figure 1.**
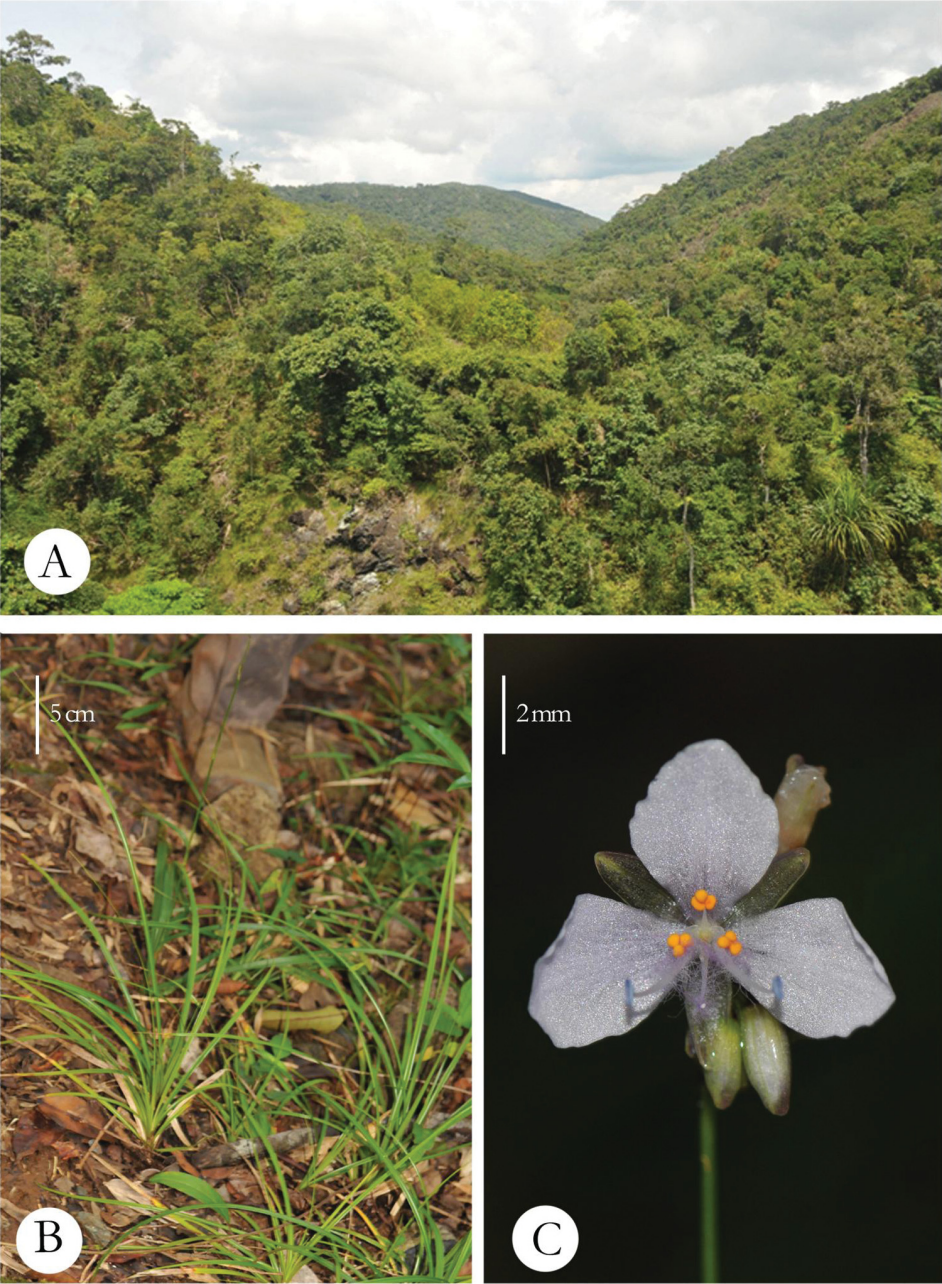
*Murdannia saddlepeakensis* **A** Habitat (a view of Saddle Peak National Park) **B** Habit **C** Flower, ventral view.

**India**. North Andaman: Saddle Peak National Park, open scrub forests (Fig. 1-A), 13°09'N, 93°01'E, at 508 m, 18 November 2011, M.V.Ramana *0550* (holotype: CAL; isotypes: US, BSI, SUK, PBL).

#### Description.

**Figure 2.**
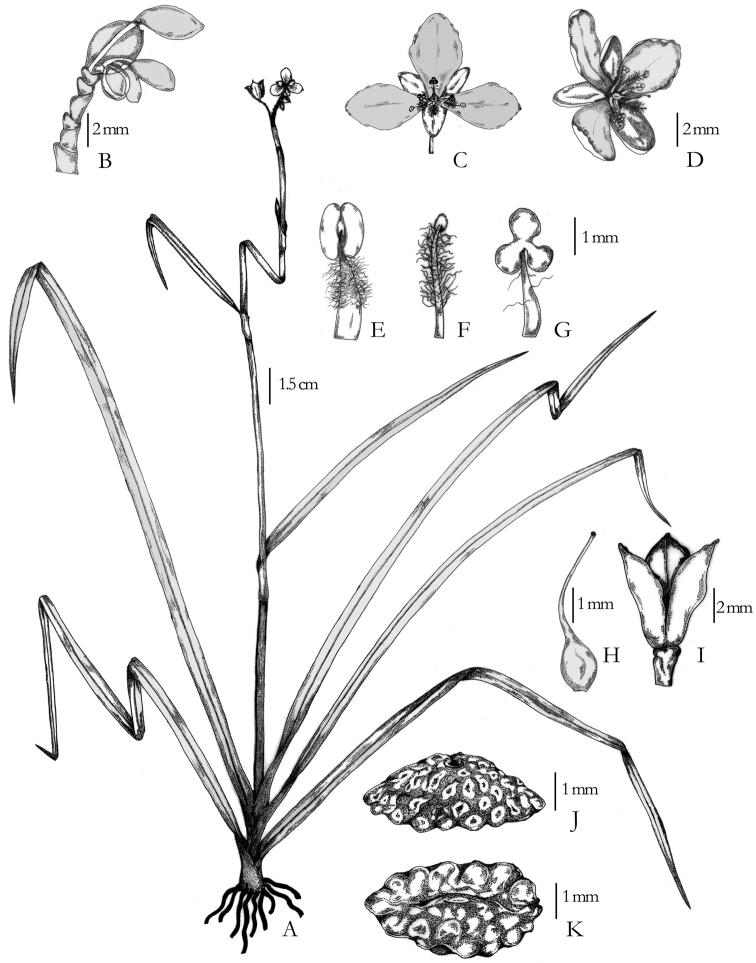
*Murdannia saddlepeakensis* **A** Habit **B** Inflorescence **C** Flower, ventral view **D** Flower, lateral view **E** Stamen **F** Rudimentary stamen **G** Staminode **H** Pistil **I** Capsule **J** Seed, lateral view **K** Seed, ventral view. All from *M V Ramana 0550*. Drawn by Mayur Nandikar

Erect, 40–60 cm high, glabrous perennial with a basal rosette of leaves (Fig. 1-B); roots fibrous, 2–4 cm long and 2 mm in diam. Leaves rosette, sheaths 0.5–1 cm long, lamina narrowly linear, 20–60 cm long, 0.4–0.8 cm wide, apex acuminate, base rounded merged into the sheath, margins entire; cauline leaves with sheaths 0.2–2 cm long, glabrous, narrowly lanceolate to linear, 1–25 cm long, 0.2–0.5 mm wide, base rounded, apex acute to acuminate, glabrous, margin entire, often scabrid; flowering shoot terminal in the basal rosette, erect, 20–40 cm long, unbranched or rarely branched from apically reduced cauline leaves (a bract). Inflorescence terminal and axillary (from uppermost foliaceous bract) of peduncled cincinni (Fig. 2–B); peduncles 2–7 cm long, glabrous, cincinni to 2 cm long, few flowered, bracteoles 5 mm long, caducous. Flowers bisexual (Fig. 1–C; Fig. 2–C, D), c. 1.5 cm wide, opening 1230–1600 hr; pedicels (2–) 3–5 mm long (not declinate in capsule); sepals elliptic to oblong elliptic, 5–6 mm long, pale white to green; petals ovate to obovate, lilac to pale lavender; stamens 2 (Fig. 2E), filaments densely bearded, (3–) 4 mm long, anthers elliptic, c. 1 mm long; staminodes 3 (Fig. 2G), antepetalous with glabrous to sparsely bearded filaments, antherodes tri-lobed, yellow; one rudimentary stamen, antisepalous (Fig. 2F) with densely bearded filament ending with sterile knob; ovary glabrous; style recurved towards staminodes, (3–)4 mm long; stigma simple. Capsule subglobose, 4–5 mm long, 3 mm wide, locules 1-seeded. Seeds (Fig. 2J, K) elliptic or rarely ovoid, 2.5–5 mm long, 1.5–3 mm wide, testa scorbiculate on all surfaces, the depressions often partially uniting on the dorsal surface, forming a little larger, irregular depressions, dark brown, hilum linear or oblong-linear, embryotega dorsal-semidorsal, farinose sparsely in all depressions and around the embryotega.

#### Distribution.

*Murdannia saddlepeakensis*is so far only known from Saddle Peak National Park, North Andaman Islands, India.

#### Ecology.

It grows in an open scrub forest in rocky situations at an elevation of 508 m. The common associates are *Sonerila andamanensis* Stapf & King (Melastomataceae), *Ophiorrhiza mungos*L.(Rubiaceae) and *Gomphostemma javanicum*(Blume)Benth. (Lamiaceae). It was observed flowering and fruiting from October to February.

**Etymology.** *Murdannia saddlepeakensis* is named after the type locality Saddle Peak National Park. It is the highest peak of the entire archipelago, reaching an altitude of 732 m.

#### Conservation status.

*Murdannia saddlepeakensis* was collected only once from the Saddle Peak National Park (North Andaman Islands). At this site c. 25 individuals in an open scrub forest in rocky situations were observed and hence it is assumed to be rare. However, larger part of the National Park is unexplored due to human inaccessibility. Therefore, the species can be accessed as “Data Deficient” (DD), using the criteria of IUCN (2001).

#### Discussion.

*Murdannia saddlepeakensis* belongs to the group *Terminatae* G. Brückn. (173: 1830) [Inflorescence terminal, many-flowered, the main shoot and lateral flowering shoots few to several, shortened, completely tufted]. In India, series *Terminatae* is represented by eight species viz. *Murdannia dimorpha* G. Brückn., *Murdannia divergens* (C.B. Clarke) G. Brückn., *Murdannia hookeri* (C.B. Clarke) G. Brückn., *Murdannia japonica* (Thunb.) Faden, *Murdannia gigantea* (Vahl) G. Brückn., *Murdannia loriformis* (Hassk.) R.S. Rao & Kammathy, *Murdannia nudiflora* (L.) Brenan and *Murdannia simplex* (Vahl) Brenan (modified after Brückn. 1930). The first four species have more than two seeds per locule and rest four have two seeds per locule while *Murdannia saddlepeakensis* has single seed per locule.

*Murdannia saddlepeakensis* closely resembles *Murdannia simplex* but can be easily distinguished by its narrow linear leaves, terminal flowering shoot in the basal rosette, glabrous leaf sheaths, single seeded locule, elliptic seed with scorbiculate surface. In addition, *Murdannia saddlepeakensis* is restricted to northern Andaman Island whereas *Murdannia simplex* is much more wide spread in Tropical Africa and Asia. *Murdannia gigantea* with a terminal flowering shoot also is similar to *Murdannia saddlepeakensis* but differs in having thick, fibrous roots, broad leaves, three stamens and seeds with dorsal embryotega. *Murdannia saddlepeakensis* can also be mistaken for the widespread *Murdannia loriformis* in general. However, the presence of erect, terminal flowering shoot and scorbiculate, single seeded locule along with anthesis by noon supports its distinctness.

Pandey and Diwakar (2008) recorded five species of *Murdannia* from Andaman & Nicobar Islands namely *Murdannia crocea* (Griff.) Faden subsp. *crocea, M. gigantea* (Vahl) G. Brückn.,* M. nudiflora* (L.) Brenan,* M. spirata* (L.) G.Brückn. and *Murdannia vaginata* (L.) G.Brückn.The occurrence of *Murdannia crocea* (Griff.) Faden subsp. *crocea* from Andaman seems to be erroneous as the screening of herbarium specimens at Kew have revealed that *Murdannia crocea* subsp. *crocea* is known only from Myanmar, Tenasserim [*Herb. Helfer 5497* (K!)]. No one has reported this species till from India after Helfer’s collection. A key for the species of the genus *Murdannia* in Andaman and Nicobar Islands and closely related taxa of the new species is given below to facilitate identification(modified after Faden 2000).

**Table:** 

1	Plants with basal rosette leaves	2
–	Plants without basal rosette leaves	5
2	Rosette leaves 0.4–0.8 cm wide; capsule subglobose; locule one seeded	*Murdannia saddlepeakensis*
–	Rosette leaves (0.5–) 0.8–1.5 cm wide; capsule ovoid to ellipsoid; locule two seeded	3
3	Flowering shoots terminal in the rosette; seeds ovoid to ellipsoid, 2.5–4 ×2–2.5 mm, hilum linear	*Murdannia gigantea*
–	Flowering shoots lateral in the rosette; seeds ovoid to obovoid, 1.6–2 × 1.5 mm, hilum linear to oblong	4
4	Pedicels 3–5 mm long; seeds 1.5–2 mm long; flowers 12–15 mm wide, opening after noon	*Murdannia simplex*
–	Pedicels 2–3 mm long; seeds 1.4–1.8 mm long; flowers 9–12 mm wide, fading by noon	*Murdannia loriformis*
5	Leaves subtending inflorescence bract-like; capsule globose; locule one seeded	*Murdannia vaginata*
–	Leaves not subtending inflorescence bract-like; capsule ovoid to obovoid or elliptic; locule more than 2 seeded	6
6	Leaves linear-lanceolate to linear-oblong; seeds 2 per locule	*Murdannia nudiflora*
–	Leaves lanceolate to ovate; seeds 3–7 per locule	*Murdannia spirata*

## Supplementary Material

XML Treatment for Murdannia saddlepeakensis
